# Three highly variable genome regions of the four dengue virus serotypes can accurately recapitulate the CDS phylogeny

**DOI:** 10.1016/j.mex.2022.101859

**Published:** 2022-09-17

**Authors:** Eduardo D. Rodríguez-Aguilar, Jesús Martínez-Barnetche, Mario H. Rodríguez

**Affiliations:** Centro de Investigación Sobre Enfermedades Infecciosas, Instituto Nacional de Salud Pública, Av. Universidad 655, Cuernavaca, Morelos C.P. 62100, Mexico

**Keywords:** Dengue virus, Phylogeny, Highly variable regions, Genotyping, Lineages, Bayesian inference

## Abstract

The circulation of the four-dengue virus (DENV) serotypes has significantly increased in recent years, accompanied by an increase in viral genetic diversity. In order to conduct disease surveillance and understand DENV evolution and its effects on virus transmission and disease, efficient and accurate methods for phylogenetic classification are required. Phylogenetic analysis of different viral genes sequences is the most used method, the envelope gene (E) being the most frequently selected target. We explored the genetic variability of the four DENV serotypes throughout their complete coding sequence (CDS) of sequences available in GenBank and used genomic regions of different variability rate to recapitulate the phylogeny obtained with the DENV CDS. Our results indicate that the use of high or low variable regions accurately recapitulate the phylogeny obtained with CDS of sequences from different DENV genotypes. However, when analyzing the phylogeny of a single genotype, highly variable regions performed better in recapitulating the distance branch length, topology, and support of the CDS phylogeny. The use of three concatenated highly variable regions was not statistically different in distance branch length and support to that obtained in CDS phylogeny.

• This study demonstrated the ability of highly variable regions of the DENV genome to recapitulate the phylogeny obtained with the full coding sequence (CDS).
• The use of genomic regions of high or low variability did not affect the performance in recapitulating the phylogeny obtained with CDS from different genotypes. However, when phylogeny was analyzed for sequences from a single genotype, highly variable regions performed better in recapitulating the distance branch length, topology, and support of the CDS phylogeny.
• The use of concatenated highly variable genome regions represent a useful option for recapitulating genome-wide phylogenies in analyses of sequences belonging to the same DENV genotype.

## Graphical abstract

**Figure:**
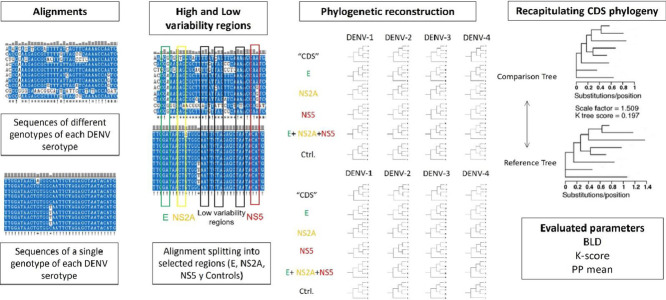

Specifications table

**Table:** 

Subject area:	Biochemistry, Genetics and Molecular Biology
More specific subject area:	Phylogenetics
Name of your method:	*Recapitulation of DENV full-CDS phylogeny by highly variable genome regions*
Name and reference of original method:	Whole-genome phylogeny
Resource availability:	*All sequences used in this study were downloaded from the Virus Pathogen Database and Analysis Resource available at:*https://www.viprbrc.org/brc/home.spg?decorator=vipr

**Method details**

## Rationale

Dengue viruses (DENV) comprise four antigenic diverse serotypes (DENV-1 to 4); mainly transmitted to humans through the bite of *Aedes* female mosquitoes that acquire the virus from a previous infected blood meal. The DENV genome is contained in single-stranded positive-sense RNA (ssRNA+) of ∼11 kb. It encodes a single open reading frame (ORF) flanked by 5’ and 3’ untranslated regions. The ORF encodes a single polyprotein which is cleaved into three structural (C: Capsid, M: membrane, E: envelope) and seven non-structural proteins (NS1, NS2A, NS2B, NS3, NS4A, NS4B, NS5) [1]. NS5 is an RNA dependent RNA polymerase, lacking proofreading activity. The errors generated by this enzyme (10-3 to 10-5 substitution per nucleotide copied per round of replication) cause the accumulation of genetically distinct variant populations [2,3].

The spread of the DENV serotypes around the world was accompanied by the accumulation of intra-serotype genetic variation and the emergence of different monophyletic groups called genotypes [4]. Each of the four serotypes comprise a number of phylogenetically distinct genotypes, which differ in geographical distribution and sometimes in the clinical manifestations produced by their infections [4]. Based on the phylogeny of the envelope gene sequences, DENV-1 has five genotypes (I–V), among which genotypes I, IV, and V are prevalent worldwide [5]. DENV-2 has six genotypes: Asian I, Asian II, Cosmopolitan, American, Asian-American and sylvatic [6]; DENV-3 has four genotypes: I, II, III, IV [7] and DENV-4 has four genotypes I-III and sylvatic [8].

Studies based on the genetic diversity of the DENV suggested the existence of lineages within each genotype, with diverse geographic and temporal relationships [9,4,6]. Recurrent introductions of DENV have been noted on multiple spatial scales, thereby limiting the persistence of a single DENV lineage in a given region [10]. Such introductions frequently cause displacement of existing clades within genotypes [11] or within serotypes [12], although co-circulation of genotypes and serotypes does occur [13]. Introduced serotypes may cause bottlenecks and displacement of previously dominant serotypes [14] significantly driving DENV diversity on a range of spatial scales [15]. Due to this pattern of lineage introduction, replacement and shift, the long-term persistence of a particular lineage in a particular locale is generally limited [16].

Although mutations occur randomly in the genome, viral proteins contain a combination of regions permissive to multiple mutations, which enable immune evasion through antigenic variation, and conserved regions with amino acid residues critical for structure and function [17]. The DENV envelope protein E- gene coding region raised more attention, because of this protein role in virus attachment and entry into the host cell, and in endosome membrane fusion, as well as target of neutralizing and enhancing antibodies [18]. However, precisely because it is the target of the evolutionary pressure exerted by neutralizing antibodies, phylogenetic analysis using genotyping of E gene sequences may not be the most accurate [19]. Other studies used a partial or truncated 5’ or 3’ C-PrM regions for DENV phylogenetic analyses in a limited number of isolates from individual countries [20], [21], [22]. A few studies, restricted to a particular region or country, recently documented the efficacy of the ORF or complete genome sequences for identifying the phylogenetic relationship of DENV-2 isolates [[23], [24], [25], [26],^19^]. However, epidemiological surveillance based on whole genomes is not feasible in many endemic countries due to the technical and analytical complexity and high costs [27]. Unfortunately, the lack of a gold standard for phylogenetic inferences makes it difficult to perform comparative evaluations of the different approaches used to discern phylogenetic relationships among DENV isolates.

In this study, we explored the genetic variability throughout the complete coding sequence (CDS) of the four DENV serotypes, and the use different genomic regions of high and low variability to recapitulate phylogeny obtained with the whole DENV genome of a sample of sequences of worldwide origin.

## Materials and methods

### Genetic variability analysis

A total of 5885 nucleotide sequences-data set (2086 of DENV-1, 1625 of DENV-2, 1516 of DENV-3 and 658 of DENV-4) including four DENV reference sequences, from the Virus Pathogen Database and Analysis Resource (ViPR) of the National Institute of Allergy and Infectious Diseases (NIH/DHHS) (accessed March 2020), were initially included in an analysis of genetic variability. For sequence's details, see supplementary file Seq_info.xlsx).

To investigate whether useful dengue phylogenetic signals could be detected in specific regions of the DENV genome (Conserved or Variable Regions) we performed a genetic variability analysis to identify candidate regions and compared their performance in replicating the DENV phylogeny to that obtained when the CDS was used.

To identify conserved and highly variable regions, 45 nt non-overlapping sliding windows of the CDS sequences of the four DENV serotypes, together and from each serotype separately, were aligned using the software Clustal Omega (v. 1.2.1.) For each window, the number of nucleotide changes was determined, and the ratio of synonymous and non-synonymous mutations was determined for each codon. The regions with the highest number of mutations and highest ratios of synonymous and non-synonymous mutations and the regions with the lowest number of mutations and the lowest rate of synonymous and non-synonymous mutations were used in subsequent phylogenetic analysis.

### Phylogenetic analysis

To investigate the sensitivity of the different regions to discriminate between sequences belonging to the same or to different genotypes, two different sequence data sets were used. The first contained sequences from different genotypes of each serotype and the second contained sequences from a single genotype of each serotype. The genotypes of the second data set were the predominant genotypes in the Americas (genotype V of DENV-1, Asian-American genotype of DENV-2, genotype III of DENV-3 and genotype II of DENV-4). To construct these data sets, the genotype of each of the 5885 ViPR sequences was determined, grouped according to genotype, and redundant sequences were removed to avoid overrepresentation of sequences due to sampling. Sequences from different genotypes were randomly selected from the remaining sequences to complete six different alignments of 30 sequences, to complete the first set of data (180 sequences). To complete the second data set (180 sequences), six alignments of 30 sequences belonging to the same genotype were constructed.

Possible sequence recombination was screened using GARD (Genetic Algorithm for Recombination Detection) [28] available in the Datamonkey web server. The multiple sequence alignments for each group, based on the Percent nucleotide identities (PNI) calculated using p-distances, were constructed using Clustal Omega (Version 1.2.1) [29].

Three highly variable regions (Hi) of 500 nt each corresponding to E, NS2A and NS5 (Hi-E, HI-NS2A and HI-NS5), and three 500 nt regions with a low rate of variability (Lo) of NS3, NS4B and NS5 (Lo-NS3, Lo-NS4B and Lo-NS5) were extracted from the generated alignments. Phylogenetic analyses were carried out using the three highly variable regions and the three lowly variable regions separately, and one concatenated sequence constructed with the three highly (Hi-Concatenated) or lowly (Lo- concatenated) variable regions, respectively.

The best-fit model of nucleotide substitution for each alignment was selected, based on the Bayesian Information Criterion (BIC), available in ModelTest 3.5 [30]. The GTR + G + I model (general time-reversible model with gamma-distributed rates of variation among sites and a proportion of invariable sites) was found to be the best-fit model for all alignments. Phylogenetic trees were constructed with these sequences using Mr. Bayes software v.3.2 [31].

### Recapitulating the phylogeny of the CDS

Using trees constructed with the complete coding region (CDS) of each group as references, we evaluated the performance of each candidate sequence (obtained from the genetic variability analysis) in recapitulating the phylogeny of the full coding sequence. For each tree constructed with the different regions to be evaluated, relative branch length, topological incongruence and tree confidence were calculated. Relative branch length was calculated as a scaling factor, using the branch length distance (BLD) [32]. This distance is sensitive to branch length similarity and it is expressed as the scale factor that approximates as much as possible the global divergence of the reference and comparison trees. The topological incongruence was estimated with the K-tree score [33], this parameter calculates the minimum branch length distance from one tree to another after scaling one of them. Thus, trees that are similar in shape to the reference tree receive a low K-tree score, whereas those that are different get a relatively higher K-tree score, regardless of their overall rates. The method that calculates the K tree score (as well as the scaling factor) was implemented in the Perl program Ktreedist Version 1.0 [33]. The tree's confidence was calculated as the mean posterior probability (mean PP), this parameter has been used to assess if the alteration of certain conditions was beneficial or detrimental for the phylogenetic reconstruction [34]. In order to normalize the value according to the nodes obtained in each group of trees, the mean PP was estimated by adding the posterior probability of all the nodes obtained in each comparison tree and dividing by the number of nodes obtained in the corresponding reference tree.

## Results (method validation)

### Genetic variability analysis

**Fig. 1:**
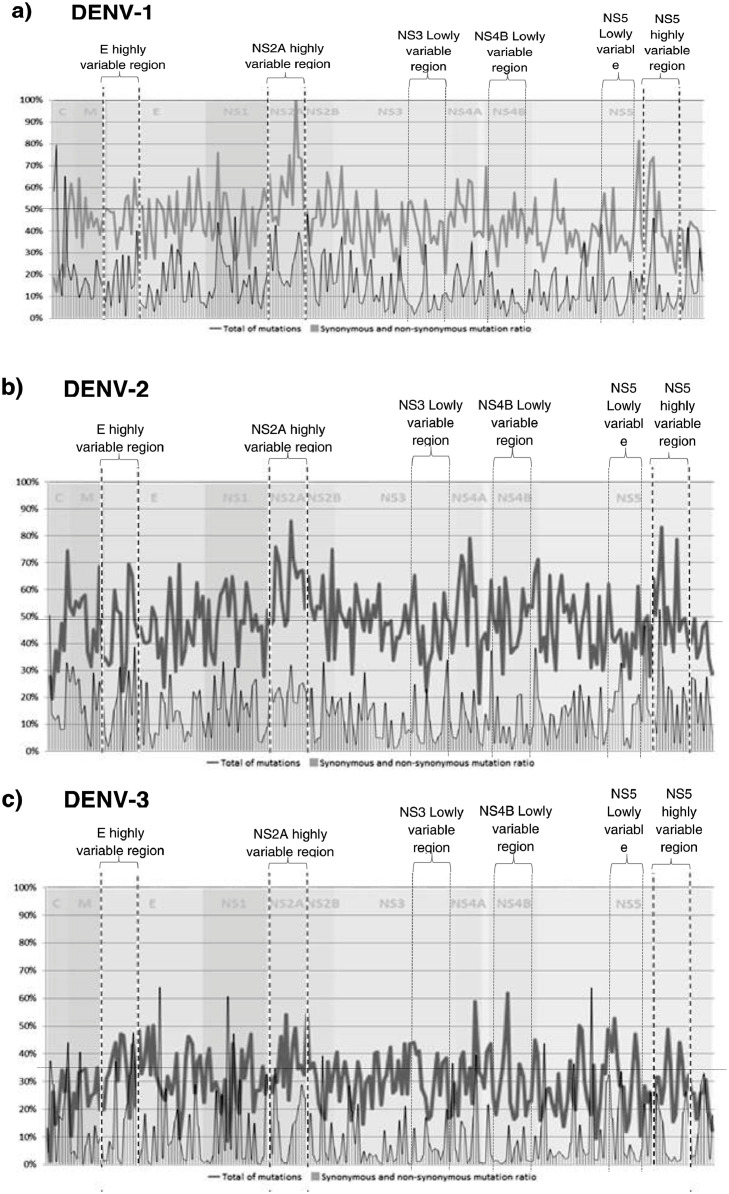

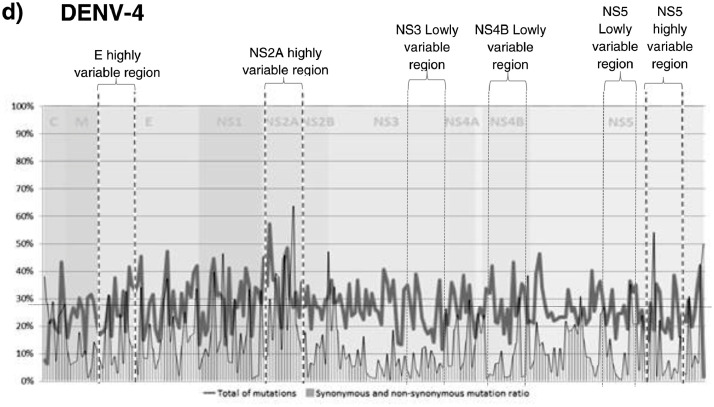
Genome-wide analysis of hypervariable sites for individual DENV serotypes (a. DENV1, b. DENV2, c. DENV3 and d DENV4). Mutation profile of nucleotides, Synonymous and non-synonymous mutation rate. The “x” axis represents the position in the DENV CDS and “y” axis represents the variability score, where 100 indicate the highest rate variability.

**Fig. 2:**
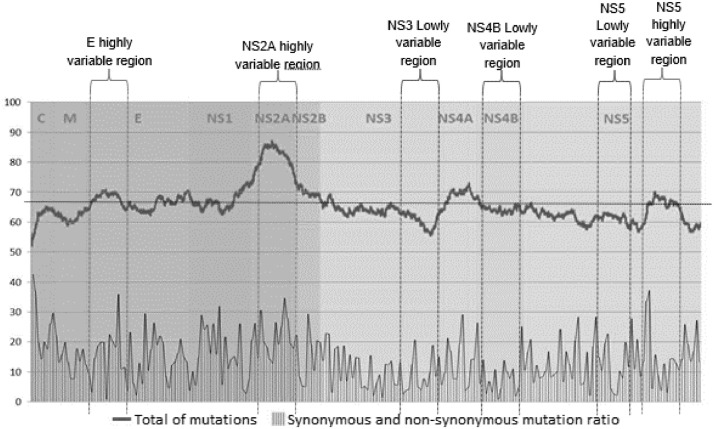
Genome-wide analysis of hypervariable sites from the four DENV serotypes together. Mutation profile of nucleotides, Synonymous and non-synonymous mutation rate. The “x” axis represents the position in the DENV CDS and “y” axis represents the variability score, where 100 indicate the highest rate variability.

The analysis of genome variability of each serotype separately identifies regions of high variability and highly conserved regions, especially for DENV 1 and 2, while DENV 3 and 4 genomes showed a less defined pattern of variation (Fig. 1). The analysis of the hypervariable regions and the proportion of synonymous and non-synonymous mutations in the four DENV serotypes together showed that the region with the highest number of mutations occurred in the NS2A followed by regions in the E proteins, NS4 and NS5 genes. Analysis of synonymous and non-synonymous mutations across the CDS showed a pattern of peaks with a high proportion of non-synonymous mutations alternating with peaks with a low proportion of non-synonymous mutations. The highest peaks of non-synonymous mutations (over 30% of the mutations found were non-synonymous) occurred in the C, E, NS2A and NS5 genes. The regions with the lowest number of mutations and the lowest rate of synonymous and non-synonymous mutations were located in NS3, NS4B and NS5 (less than 10% of the mutations found were non-synonymous) (Fig. 2). For phylogenetic analyzes, we used the three regions of the genome with the highest number of mutations and the highest proportion of non-synonymous mutations, separately and in a sequence formed by concatenating the same three regions. These regions included a region that comprises coding sequences of domains I and II of protein E, most of the NS2A protein, except for its amino terminal, and the catalytic region of RNA polymerase corresponding to the NS5 protein (Hi-E, HI-NS2A and HI-NS5 regions) (Fig. 2). In addition, we use the three regions with the lowest number of mutations and lowest proportion of non-synonymous mutations, individually and concatenated (regions of the genome with less than 10% of non-synonymous mutations). These regions included the coding sequences of the C-terminal region of NS3 protein, most of the NS4B protein and the region between the mRNA cap domain and the RNA polymerase domain of the NS5 protein (Lo-NS3, Lo-NS4B and Lo-NS5 regions) (Fig. 2).

### Recapitulating the phylogeny of the CDS

#### Branches of the trees constructed with highly variable regions are longer and more accurate

Relative branch length distance is sensitive to branch length similarity and was calculated as the scaling factor that most closely approximates the overall divergence between the reference and compared trees. Since this is a scale, a value of 1 indicates that the overall branch distances of the reference and comparison trees are equal. Values greater than 1 indicate that the branches of the compared tree are shorter than those of the reference tree; and values less than 1 indicate that the branches of the compared trees are longer than those of the reference tree.

**Fig. 3:**
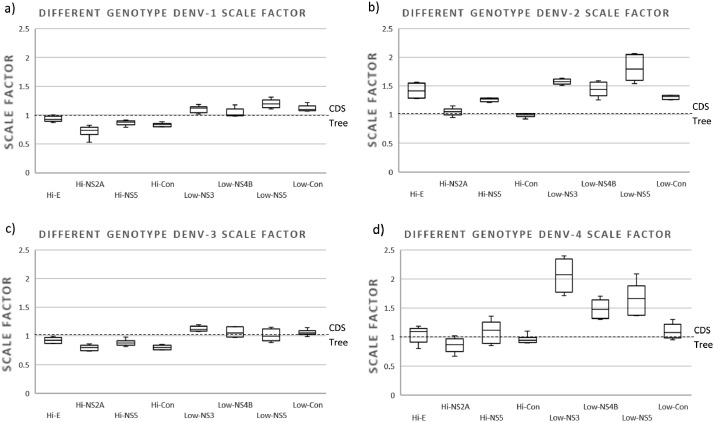
Box plot of the relative branch length of the trees constructed with sequences of different genotypes of (a) DENV-1, (b) DENV-2, (c) DENV-3, and (d) DENV-4. The trees constructed with the different regions (Hi- and Low-variables) were compared with the one constructed with their respective CDS to obtain the scaling factor. For each column, the box represents the second (Q2) and third (Q3) quartiles of the data distribution, the line within the box marks the median, and whiskers denote the most extreme data (that are not outliers).

In the analysis of the branch length distances, we observed that the highly variable regions of the four DENV serotypes obtained lower scale factors compared to that of the low variable regions. This indicates that the branches of the trees constructed with highly variable regions are longer than those constructed with low variable regions. The scaling factor values of the trees constructed with highly variable regions were very close to 1 or below 1, while the scaling factor values of the trees constructed with low variable regions were always above 1 and, in some trees, above 2. Scale factor values were more accurate and less dispersed in serotype 2 and 4 trees constructed with highly variable regions, indicating that they are more suitable for reproducing CDS phylogeny compared to low variable regions. There were no significant differences in accuracy or dispersion in serotypes 1 and 3 trees (Fig. 3).

DENV-1 and DENV-3 trees constructed with all highly variable regions produced trees with longer branches (values slightly below 1); while those constructed with the low variable regions produced trees with shorter branches than the reference tree (values slightly greater than 1) (Fig. 3a and c). For DENV-2, all regions evaluated produced trees with shorter branches than the reference one, with the exception of the NS2A highly variable region (Hi-NS2A) and the highly variable concatenated (Hi-Con) regions, whose relative branch distances were not statistically different from those of the reference tree (*p*-values Hi-NS2A = 0.9952 and Hi-Con = 1) (Fig. 3b and Table S1). For DENV-4, which had the highest data variability of the four serotypes, the low variable regions were inaccurate, and all the trees obtained had far shorter branches than those of the reference (scale factor values between 1.3 and 2.4), with the exception of a few trees constructed with concatenated low variable regions (Low-Con) (scale factor values between 1 and 1.3). The highly variable regions obtained more accurate and less dispersed scaling factors (scale factor values between 0.7 and 1.4), with the exception of the concatenated highly variable regions (Hi-Con), whose values approached 0.55 (Fig. 3d).

**Fig. 4:**
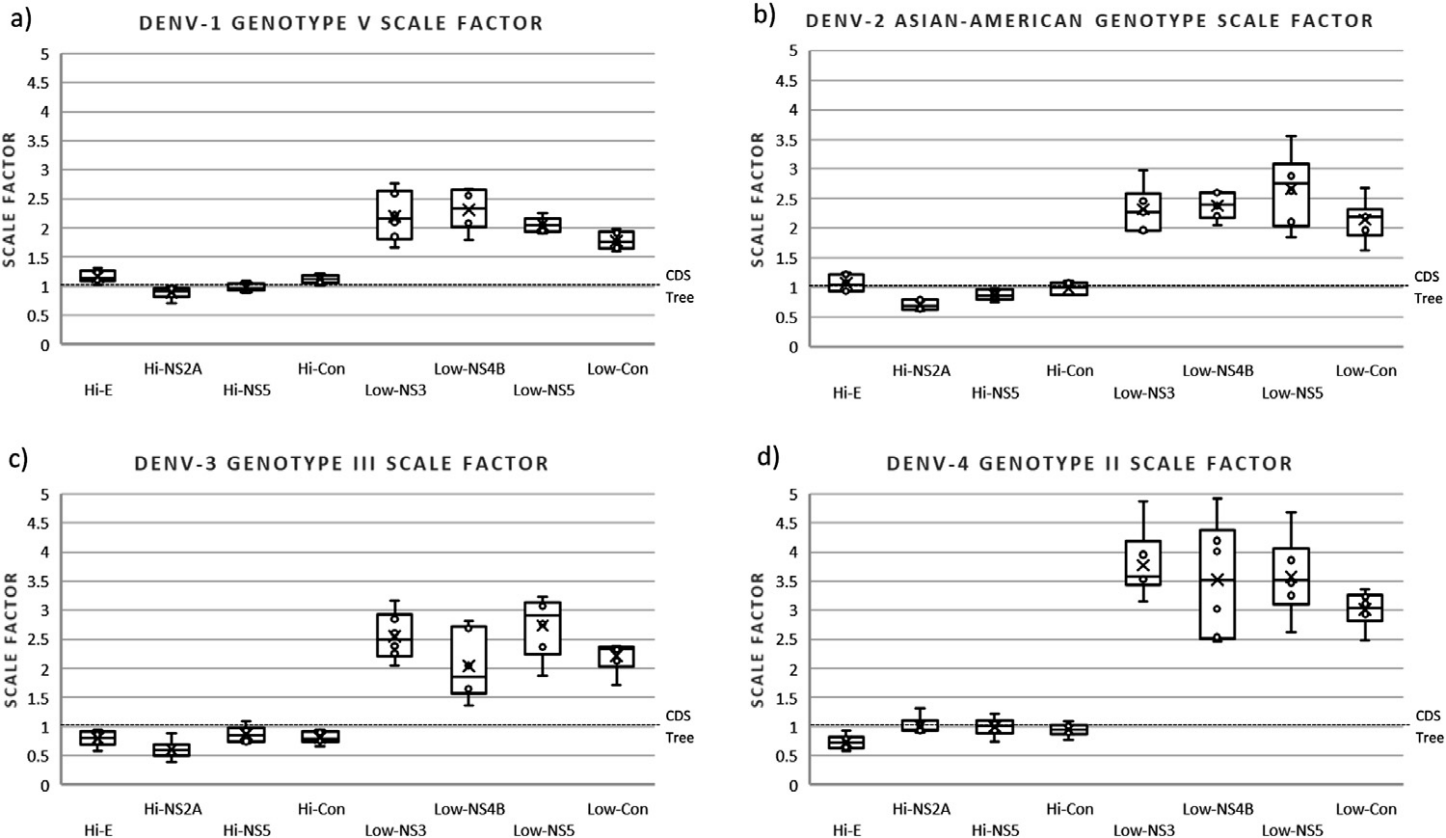
Box plot of the relative branch length of the trees constructed with sequences of a single genotype of (a) DENV-1 (genotype V), (b) DENV-2 (genotype Asian-American), (c) DENV-3 (Genotype III), and (d) DENV-4 (genotype II). The trees constructed with the different regions (Hi- and Low-variables) were compared with the one constructed with their respective CDS to obtain the scaling factor. For each column, the box represents the second (Q2) and third (Q3) quartiles of the data distribution, the line within the box marks the median, whiskers denote the most extreme data (that are not outliers), and circles indicate outliers.

When assessing BLD in sequences of the same genotype (less divergent), we found more notable differences between regions of high and low variability. All trees constructed with all highly variable regions produced trees with branches similar to the reference tree (scaling factor values between 0.5 and 1.3); while those constructed with the low variable regions produced trees with branches far shorter than the reference tree (scaling factor values between 1.3 and 4.9) (Fig. 4). There was no significant difference between the BLD of the trees constructed with the highly variable regions and the BLD of the reference tree (All *p*-values > 0.3), with the exception of the BLD of the tree constructed with Hi-NS2A for DENV-2 (*p*-value = 0.037). All BLDs of trees constructed with low variable regions were significantly shorter (all *p*-values < 0.001).

#### Concatenated regions produced topologically more similar trees to that of the CDS trees than individual regions

The topological incongruence was estimated with the K-tree score, which calculates the minimum branch length distance from one tree to another after scaling one of them. Thus, a K-Score of 0 means that there is no topological incongruence between the compared trees, i.e. they are topologically identical. Trees that are similar in shape to the reference tree receive a low K-tree score whereas those that are different get a relatively higher K-tree score.

**Fig. 5:**
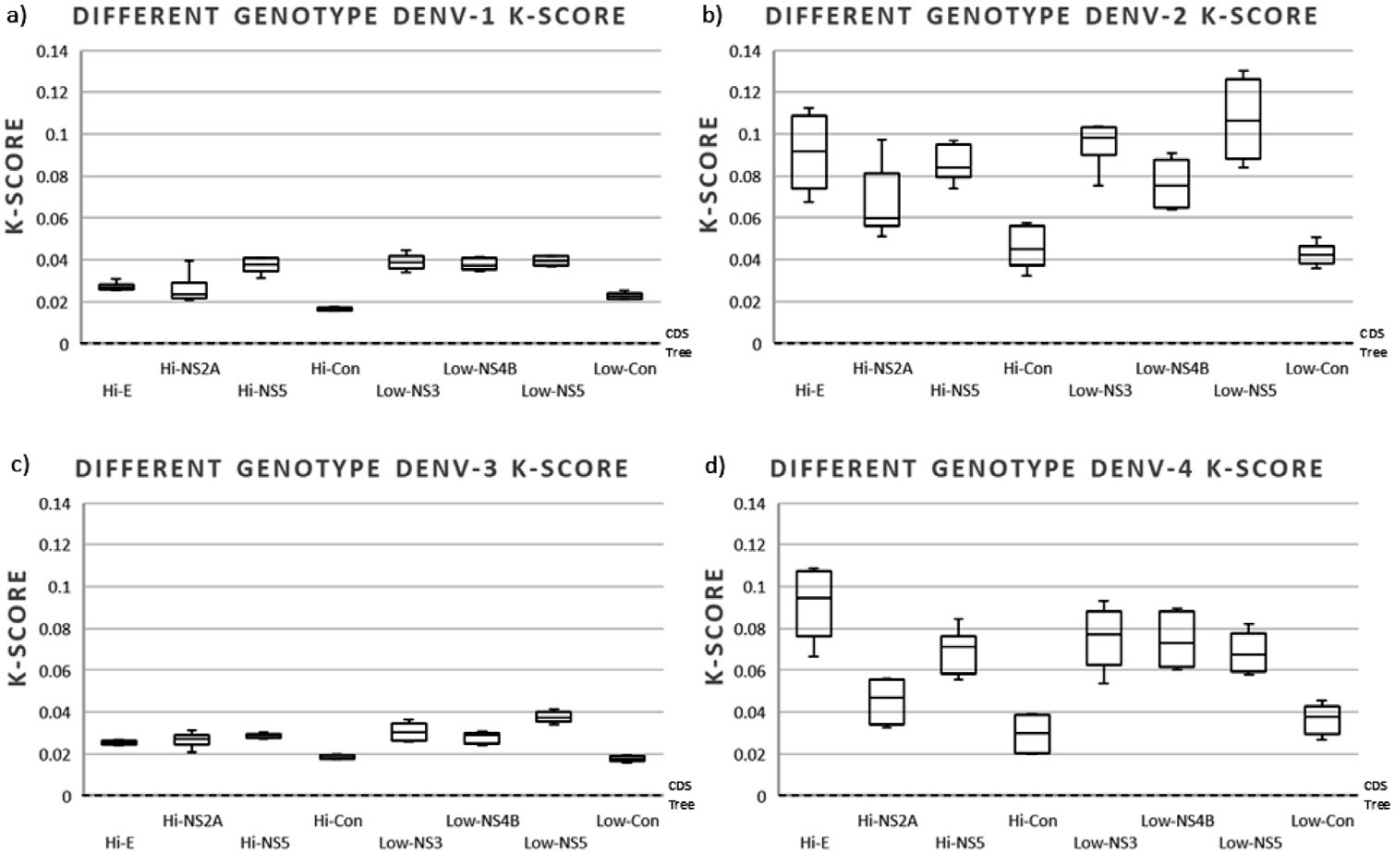
Box plot of the topological incongruence of the trees constructed with sequences of different genotypes of (a) DENV-1, (b) DENV-2, (c) DENV-3, and (d) DENV-4. The trees constructed with the different regions (Hi- and Low-variables) were compared with the one constructed with their respective CDS to obtain the K-Score. For each column, the box represents the second (Q2) and third (Q3) quartiles of the data distribution, the line within the box marks the median, and whiskers denote the most extreme data (that are not outliers).

The topological incongruence analysis estimated with the K-tree score indicated no significant differences between trees constructed with highly and low variable regions were observed for the four DENV serotypes, using sequences from different genotypes (*p*-values > 0.05). However, significant differences were observed between trees constructed with individual versus concatenated regions (*p*-values < 0.05) (Table S3). Trees constructed with concatenated regions were topologically more similar to the CDS tree and more accurate than trees constructed with individual regions for all serotypes (K-score between 0.018 and 0.045). The K-Score values for serotypes 1 and 3 were less dispersed compared to those of serotypes 2 and 4, indicating higher accuracy for DENV-1 and DENV-3 trees (Fig. 5).

**Fig. 6:**
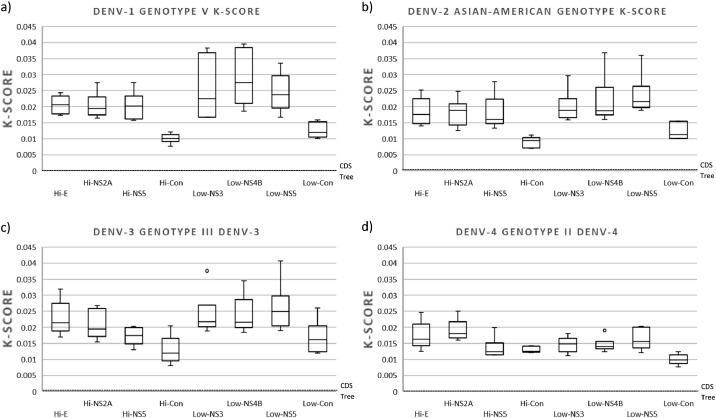
Box plot of the topological incongruence of the trees constructed with sequences of a single genotype of (a) DENV-1 (genotype V), (b) DENV-2 (genotype Asian-American), (c) DENV-3 (Genotype III), and (d) DENV-4 (genotype II). The trees constructed with the different regions (Hi- and Low-variables) were compared with the one constructed with their respective CDS to obtain the K-Score. For each column, the box represents the second (Q2) and third (Q3) quartiles of the data distribution, the line within the box marks the median, whiskers denote the most extreme data (that are not outliers), and circles indicate outliers.

Using sequences of the same genotype, we found significant differences comparing individual versus concatenated regions (*p*-values < 0.05) (Table S4). The concatenated regions obtained more topologically similar trees to the CDS tree than individual regions for all serotypes (Fig. 6), except for DENV-4 which only showed significant differences for the lower variable concatenated regions (Low-Con) (*p*-values < 0.02) (Fig. 6d). We also observed significant differences between individual high variable and low variable regions only for the E and NS2A high variable regions (Hi-E and Hi-NS2A) in DENV-1 and the NS5 high variable region (Hi-NS5) in NS3, which obtained more accurate and topologically similar trees to the CDS tree than the low variable regions individually (Fig. 6a and c).

#### Highly variable regions produce more confident trees than lower variable regions for all serotypes in the same genotype trees

**Fig. 7:**
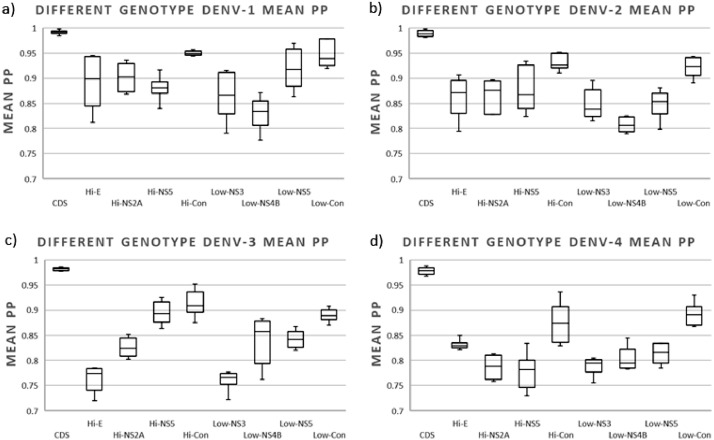
Box plot of the confidence of the trees constructed with sequences of different genotypes of (a) DENV-1, (b) DENV-2, (c) DENV-3, and (d) DENV-4. The tree confidence was calculated as mean posterior probability (mean PP) estimated by adding the posterior probability of all the nodes obtained in each comparison tree and dividing by the number of nodes obtained in the corresponding reference tree. For each column, the box represents the second (Q2) and third (Q3) quartiles of the data distribution, the line within the box marks the median, and whiskers denote the most extreme data (that are not outliers).

The tree confidence calculated as mean posterior probability (mean PP) analysis. Since not all trees recovered the same number of nodes, and in order to normalize the values according to the nodes obtained in each tree group, the mean PP was estimated by adding the posterior probability of all the nodes obtained in each compared tree and dividing it by the number of nodes obtained in the corresponding reference tree. We found no significant differences between highly variable and low variable regions of the four DENV serotypes using sequences from different genotypes (*p*-values > 0.05). However, significant differences were observed when comparing individual versus concatenated regions (*p*-values < 0.05) (Table S5). The concatenated regions produced more confident trees than individual regions for all serotypes (Fig. 7).

For DENV-1, trees constructed with all individual regions obtained values greater than 0.8. The trees constructed with concatenated regions (Hi-Con and Low Con) obtained the most confident trees with values close to 0.95 (Fig. 7a and Table S7a). This was similar for DENV-2 trees, all regions (Hi-Con and Low Con) obtained values greater than 0.8 and produced the most confident trees with values close to 0.93 (Fig. 7b and Table S7b). Differences according to the individual region used were observed among trees of DENV-3. Hi-E and Low-NS3 produced the least confident trees (Fig. 7b and Table S7a). The Hi-Con and Hi-Low obtained values over 0.87 (Fig. 7c and Table S7a). The least confident trees among the four serotypes were those of DENV4, where the best performing individual region was Hi-E with values close to 0.83 and the concatenated regions were Hi-Con and Low-con regions obtained values over 0.91 and 0.89, respectively (Fig. 7d and Table S7a).

**Fig. 8:**
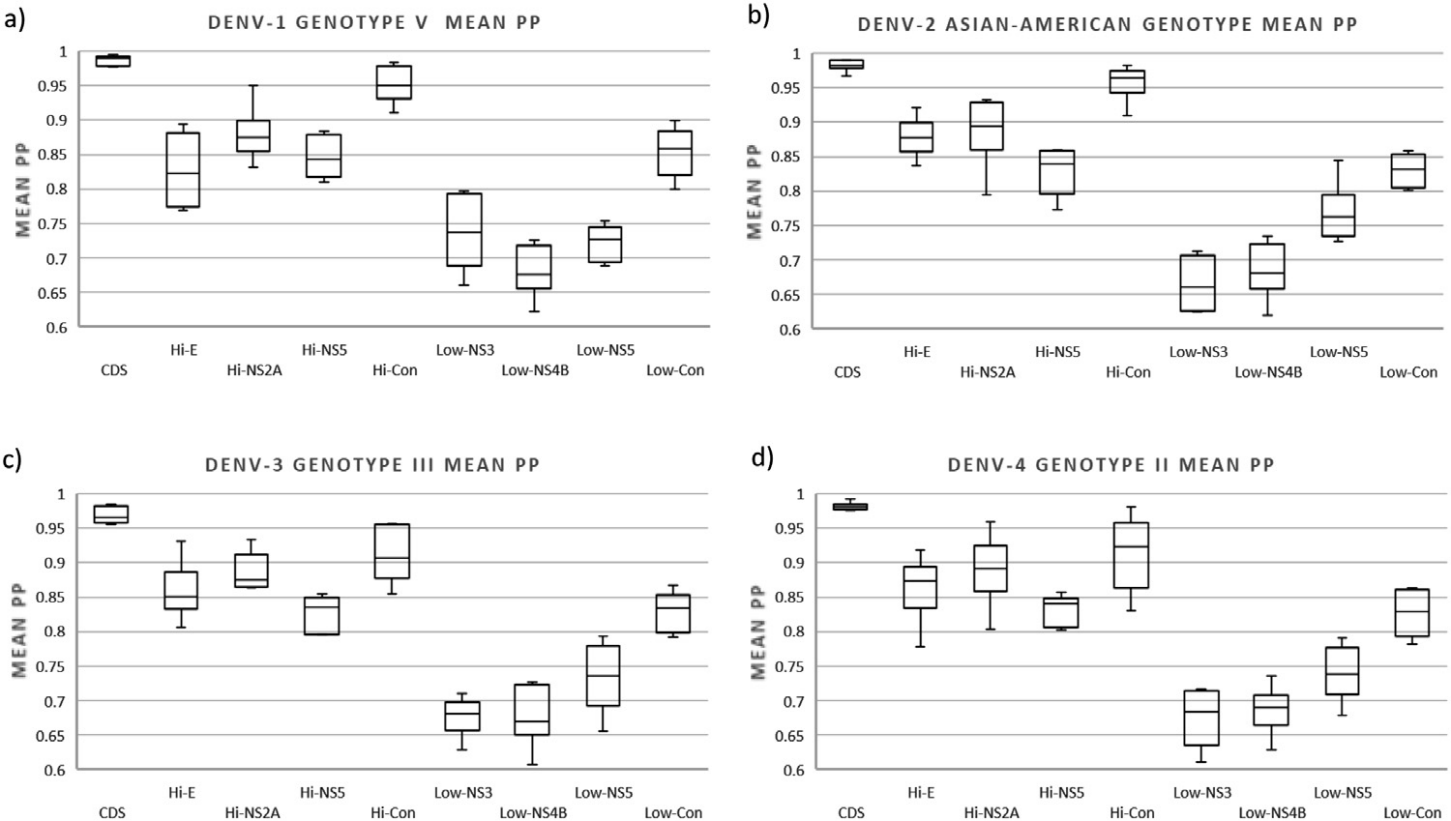
Box plot of the confidence of the trees constructed with sequences of a single genotype of (a) DENV-1 (genotype V), (b) DENV-2 (genotype Asian-American), (c) DENV-3 (Genotype III), and (d) DENV-4 (genotype II). The tree confidence was calculated as mean posterior probability (mean PP) estimated by adding the posterior probability of all the nodes obtained in each comparison tree and dividing by the number of nodes obtained in the corresponding reference tree. For each column, the box represents the second (Q2) and third (Q3) quartiles of the data distribution, the line within the box marks the median, and whiskers denote the most extreme data (that are not outliers).

Using sequences of the same genotype, we found significant differences in tree confidence between highly variable or lower variable regions and whether used individually or concatenated. Highly variable regions obtained more confident trees than lower variable regions for all serotypes (mean PP higher than 0.76) and the highly variable regions concatenated obtained more confident trees than regions individually (mean PP higher than 0.84) (Fig. 8).

For DENV-1, the highly variable concatenated regions obtained the most confident trees with values over 0.89, trees with lower variable regions obtained the less confident trees with values below 0.79 (Fig. 8a and Table S7b). For DENV-2, the highly variable concatenated regions obtained the most confident trees with values over 0.9, individual lower variable regions obtained the less confident trees with values between below 0.84 (Fig. 8b and Table S7b). For DENV-3, the highly variable concatenated regions obtained the most confident trees with values over 0.89, individual lower variable regions obtained the less confident trees with values between below 0.78 (Fig. 8c and Table S7b). For DENV-4, the highly variable concatenated regions obtained the most confident trees with values over 0.86, individual lower variable regions obtained the less confident trees with values below 0.78 (Fig. 8d and Table S7b).

## Discussion

Evolutionary analyses of DENV genes and genomes fall under the fields of phylogenetics and phylogenomics in which phylogenetic trees (phylograms) are used to infer the relationship between viruses and how they evolve over time [35]. As DENV evolution occurs on a scale, which often matches that of their transmission, the genetic differences between DENV strains transmitted during epidemics are generally detectable within weeks, especially when using whole virus genomes [36]. However, epidemiological surveillance based on complete genomes, is not feasible in many endemic countries due to the analytical complexity and high costs of sequencing [27]. The current understanding of the molecular epidemiology and evolution of DENV is significantly limited by a major deficit of sequence data from endemic developing countries (less than 1% of all global DENV envelope sequence data coming from African countries) [37]. This study proposes short consensus genomic regions to reproduce phylogenies based on the complete coding sequence under different conditions.

The best criteria for choosing loci to address a particular phylogenetic question are still under debate, but the most commonly cited attribute is their evolutionary rate [38], [39], [40]. If a gene evolves too slowly with respect to a particular split in the tree, it will show too few differences to provide enough information to correctly infer the possible relationships. If it evolves too fast, different mutations at the same site cannot be distinguished and the gene loses its discriminatory power. Our results showed that DENV genome has patterns of nucleotide variations with regions of high variability and highly conserved regions. The regions with the greatest variability occur in genes coding the E, NS2A and NS5 proteins and that the regions with the lowest variability occur in those coding the NS3, NS4B and NS5 proteins. This genetic variability pattern was previously documented [41].

Using trees constructed with the CDS as reference trees, we found that highly variable regions produced trees with longer branches and lower variable regions produced trees with shorter branches. This was expected since branch length indicates genetic change, i.e., the longer the branch, the more genetic change (mutations) has occurred. However, this did not occur for trees constructed with sequences from different DENV-2 genotypes, where all regions evaluated produced shorter branches than the reference tree. Except for the highly variable NS2A region (Hi-NS2A) and the Hi-Con, which had branch lengths very similar to those on the reference tree (Fig. 3b). This may reflect high levels of variation within dengue serotype 2 outside the regions evaluated here. In contrast to the other serotypes, evidence of positive selection has been detected in the NS2B and NS5 genes [42] and positive selection has been detected in the E protein gene for the Asiatic I and Cosmopolitan genotypes [43].

Another interesting result was that in trees of less divergent sequences (sequences of the same genotype), the performance of the lower variable regions in reproducing the CDS phylogeny decreased considerably. This can be explained by the fact that in this situation, the lower variable sequences produced trees with very short branches. Short branches may simply indicate that the virus had not enough time to accumulate substitutions and are difficult to resolve because there is too little informative variation, resulting in inconsistent gene histories [44].

This limitation of the low variable regions in resolving phylogenies was reflected in the support of the trees. The mean support of the trees from the low variable regions were statistically lower than those obtained from the highly variable regions. Likewise, the concatenated regions obtained higher values than the individual regions. Concatenated datasets offer two major advantages. First, the confidence in the inferred relationships is greatly improved when they are supported by different genes information. Second, each individual gene may not contain sufficient information to resolve all relationships; therefore, combining gene data may increase the amount of phylogenetic signal and provide the necessary resolution.

## Conclusion

Our results indicate that the use of high or low variable genome regions did not affect the performance in the recapitulation of the phylogeny obtained with CDS of different genotypes. However, when analyzing the phylogeny of a single genotype, highly variable regions performed better in recapitulating the distance branch length, topology, and support of the CDS phylogeny. The use of three concatenated highly variable regions may represent a useful option for recapitulating whole genome phylogenies in analyses of sequences belonging to the same DENV genotype.

## CRediT authorship contribution statement

**Eduardo D. Rodríguez-Aguilar:** Conceptualization, Methodology, Software, Validation, Formal analysis, Investigation, Data curation, Writing – original draft, Writing – review & editing, Visualization, Supervision, Project administration. **Jesús Martínez-Barnetche:** Conceptualization, Software, Formal analysis, Writing – review & editing, Visualization, Supervision. **Mario H. Rodríguez:** Conceptualization, Formal analysis, Investigation, Resources, Writing – original draft, Writing – review & editing, Visualization, Supervision, Project administration, Funding acquisition.

## Data Availability

Data will be made available on request.

## Appendix Supplementary materials

Image, application 1
