# Supplementary material for: Three highly variable genome regions of the four dengue virus serotypes can accurately recapitulate the CDS phylogeny
Source: MethodsX. 2022 Sep 17;9:101859. doi: 10.1016/j.mex.2022.101859 (PMC9516459; doi:10.1016/j.mex.2022.101859)
Supplement: Supplementary file 1 [file mmc1.docx]

**Supplementary material *and/or* additional information [OPTIONAL]**

Table S1

| **DENV-1** | **P-value** | **CDS** | **Hi-E** | **Hi-NS2A** | **Hi-NS5** | **Hi-Con** | **Low-NS3** | **Low-NS4B** | **Low-NS5** | **Low-Con** |
| --- | --- | --- | --- | --- | --- | --- | --- | --- | --- | --- |
|  | **CDS** |  | 0.65894 | < 0.001 | 0.01883 | 0.00134 | 0.14407 | 0.98191 | < 0.001 | 0.06182 |
|  | **Hi-E** | 0.65894 |  | < 0.001 | 0.68573 | 0.18074 | 0.00095 | 0.12781 | < 0.001 | 0.00029 |
|  | **Hi-NS2A** | < 0.001 | < 0.001 |  | 0.00488 | 0.05675 | < 0.001 | < 0.001 | < 0.001 | < 0.001 |
|  | **Hi-NS5** | 0.01883 | 0.68573 | 0.00488 |  | 0.99213 | < 0.001 | 0.00091 | < 0.001 | < 0.001 |
|  | **Hi-Con** | 0.00134 | 0.18074 | 0.05675 | 0.99213 |  | < 0.001 | < 0.001 | < 0.001 | < 0.001 |
|  | **Low-NS3** | 0.14407 | 0.00095 | < 0.001 | < 0.001 | < 0.001 |  | 0.69454 | 0.23062 | 0.99999 |
|  | **Low-NS4B** | 0.98191 | 0.12781 | < 0.001 | 0.00091 | < 0.001 | 0.69454 |  | 0.00235 | 0.45337 |
|  | **Low-NS5** | < 0.001 | < 0.001 | < 0.001 | < 0.001 | < 0.001 | 0.23062 | 0.00235 |  | 0.42788 |
|  | **Low-Con** | 0.06182 | 0.00029 | < 0.001 | < 0.001 | < 0.001 | 0.99999 | 0.45337 | 0.42788 |  |
| **DENV-2** | **P-value** | **CDS** | **Hi-E** | **Hi-NS2A** | **Hi-NS5** | **Hi-Con** | **Low-NS3** | **Low-NS4B** | **Low-NS5** | **Low-Con** |
|  | **CDS** |  | < 0.001 | 0.99520 | 0.00193 | 1.00000 | < 0.001 | < 0.001 | < 0.001 | 0.00018 |
|  | **Hi-E** | < 0.001 |  | < 0.001 | 0.16821 | < 0.001 | 0.17883 | 0.99999 | < 0.001 | 0.56692 |
|  | **Hi-NS2A** | 0.99520 | < 0.001 |  | 0.02182 | 0.97653 | < 0.001 | < 0.001 | < 0.001 | 0.00249 |
|  | **Hi-NS5** | 0.00193 | 0.16821 | 0.02182 |  | 0.00093 | < 0.001 | 0.07898 | < 0.001 | 0.99768 |
|  | **Hi-Con** | 1.00000 | < 0.001 | 0.97653 | 0.00093 |  | < 0.001 | < 0.001 | < 0.001 | < 0.001 |
|  | **Low-NS3** | < 0.001 | 0.17883 | < 0.001 | < 0.001 | < 0.001 |  | 0.33511 | 0.00602 | 0.00085 |
|  | **Low-NS4B** | < 0.001 | 0.99999 | < 0.001 | 0.07898 | < 0.001 | 0.33511 |  | < 0.001 | 0.35231 |
|  | **Low-NS5** | < 0.001 | < 0.001 | < 0.001 | < 0.001 | < 0.001 | 0.00602 | < 0.001 |  | < 0.001 |
|  | **Low-Con** | 0.00018 | 0.56692 | 0.00249 | 0.99768 | < 0.001 | 0.00085 | 0.35231 | < 0.001 |  |
| **DENV-3** | **P-value** | **CDS** | **Hi-E** | **Hi-NS2A** | **Hi-NS5** | **Hi-Con** | **Low-NS3** | **Low-NS4B** | **Low-NS5** | **Low-Con** |
|  | **CDS** |  | 0.50847 | < 0.001 | 0.05608 | < 0.001 | 0.03016 | 0.73386 | 0.99999 | 0.80385 |
|  | **Hi-E** | 0.50847 |  | 0.02101 | 0.96353 | 0.02982 | < 0.001 | 0.01235 | 0.30371 | 0.01743 |
|  | **Hi-NS2A** | < 0.001 | 0.02101 |  | 0.28638 | 1.00000 | < 0.001 | < 0.001 | < 0.001 | < 0.001 |
|  | **Hi-NS5** | 0.05608 | 0.96353 | 0.28638 |  | 0.35621 | < 0.001 | < 0.001 | 0.02304 | < 0.001 |
|  | **Hi-Con** | < 0.001 | 0.02982 | 1.00000 | 0.35621 |  | < 0.001 | < 0.001 | < 0.001 | < 0.001 |
|  | **Low-NS3** | 0.03016 | < 0.001 | < 0.001 | < 0.001 | < 0.001 |  | 0.72017 | 0.07164 | 0.64204 |
|  | **Low-NS4B** | 0.73386 | 0.01235 | < 0.001 | < 0.001 | < 0.001 | 0.72017 |  | 0.90260 | 1.00000 |
|  | **Low-NS5** | 0.99999 | 0.30371 | < 0.001 | 0.02304 | < 0.001 | 0.07164 | 0.90260 |  | 0.94081 |
|  | **Low-Con** | 0.80385 | 0.01743 | < 0.001 | < 0.001 | < 0.001 | 0.64204 | 1.00000 | 0.94081 |  |
| **DENV-4** | **P-value** | **CDS** | **Hi-E** | **Hi-NS2A** | **Hi-NS5** | **Hi-Con** | **Low-NS3** | **Low-NS4B** | **Low-NS5** | **Low-Con** |
|  | **CDS** |  | 0.99997 | 0.91063 | 0.99133 | 0.99998 | < 0.001 | 0.00100 | < 0.001 | 0.98933 |
|  | **Hi-E** | 0.99997 |  | 0.70342 | 0.99989 | 0.99594 | < 0.001 | 0.00362 | < 0.001 | 0.99983 |
|  | **Hi-NS2A** | 0.91063 | 0.70342 |  | 0.38862 | 0.98812 | < 0.001 | < 0.001 | < 0.001 | 0.37134 |
|  | **Hi-NS5** | 0.99133 | 0.99989 | 0.38862 |  | 0.92520 | < 0.001 | 0.01510 | < 0.001 | 1.00000 |
|  | **Hi-Con** | 0.99998 | 0.99594 | 0.98812 | 0.92520 |  | < 0.001 | < 0.001 | < 0.001 | 0.91587 |
|  | **Low-NS3** | < 0.001 | < 0.001 | < 0.001 | < 0.001 | < 0.001 |  | < 0.001 | 0.01097 | < 0.001 |
|  | **Low-NS4B** | 0.00100 | 0.00362 | < 0.001 | 0.01510 | < 0.001 | < 0.001 |  | 0.75259 | 0.01639 |
|  | **Low-NS5** | < 0.001 | < 0.001 | < 0.001 | < 0.001 | < 0.001 | 0.01097 | 0.75259 |  | < 0.001 |
|  | **Low-Con** | 0.98933 | 0.99983 | 0.37134 | 1.00000 | 0.91587 | < 0.001 | 0.01639 | < 0.001 |  |

**Table S1.** Results of the p-value of the Tukey's one-way ANOVA test on BLD of the trees constructed with sequences of different genotypes. One-way analysis of variance (one-way ANOVA) and the p-value of Tukey's test was observed to verify the existence of statistical differences between the scale factor of the different regions evaluated in recapitulating the CDS phylogeny of sequences of different genotypes. The p-values > 0.05 are shaded in gray.

Table S2

| **DENV-1** | **P-value** | **CDS** | **Hi-E** | **Hi-NS2A** | **Hi-NS5** | **Hi-Con** | **Low-NS3** | **Low-NS4B** | **Low-NS5** | **Low-Con** |
| --- | --- | --- | --- | --- | --- | --- | --- | --- | --- | --- |
|  | **CDS** |  | 0.90962 | 0.98979 | 1.00000 | 0.98459 | < 0.001 | < 0.001 | < 0.001 | < 0.001 |
|  | **Hi-E** | 0.90962 |  | 0.37337 | 0.85022 | 0.99999 | < 0.001 | < 0.001 | < 0.001 | < 0.001 |
|  | **Hi-NS2A** | 0.98979 | 0.37337 |  | 0.99678 | 0.60031 | < 0.001 | < 0.001 | < 0.001 | < 0.001 |
|  | **Hi-NS5** | 1.00000 | 0.85022 | 0.99678 |  | 0.96428 | < 0.001 | < 0.001 | < 0.001 | < 0.001 |
|  | **Hi-Con** | 0.98459 | 0.99999 | 0.60031 | 0.96428 |  | < 0.001 | < 0.001 | < 0.001 | < 0.001 |
|  | **Low-NS3** | < 0.001 | < 0.001 | < 0.001 | < 0.001 | < 0.001 |  | 0.99074 | 0.94778 | 0.02436 |
|  | **Low-NS4B** | < 0.001 | < 0.001 | < 0.001 | < 0.001 | < 0.001 | 0.99074 |  | 0.46189 | 0.00168 |
|  | **Low-NS5** | < 0.001 | < 0.001 | < 0.001 | < 0.001 | < 0.001 | 0.94778 | 0.46189 |  | 0.35521 |
|  | **Low-Con** | < 0.001 | < 0.001 | < 0.001 | < 0.001 | < 0.001 | 0.02436 | 0.00168 | 0.35521 |  |
| **DENV-2** | **P-value** | **CDS** | **Hi-E** | **Hi-NS2A** | **Hi-NS5** | **Hi-Con** | **Low-NS3** | **Low-NS4B** | **Low-NS5** | **Low-Con** |
|  | **CDS** |  | 0.99993 | 0.03700 | 0.99740 | 1.00000 | < 0.001 | < 0.001 | < 0.001 | < 0.001 |
|  | **Hi-E** | 0.99993 |  | 0.04030 | 0.94698 | 0.99983 | < 0.001 | < 0.001 | < 0.001 | < 0.001 |
|  | **Hi-NS2A** | 0.03700 | 0.04030 |  | 0.98296 | 0.73633 | < 0.001 | < 0.001 | < 0.001 | < 0.001 |
|  | **Hi-NS5** | 0.99740 | 0.94698 | 0.98296 |  | 0.99852 | < 0.001 | < 0.001 | < 0.001 | < 0.001 |
|  | **Hi-Con** | 1.00000 | 0.99983 | 0.73633 | 0.99852 |  | < 0.001 | < 0.001 | < 0.001 | < 0.001 |
|  | **Low-NS3** | < 0.001 | < 0.001 | < 0.001 | < 0.001 | < 0.001 |  | 0.99998 | 0.49541 | 0.97895 |
|  | **Low-NS4B** | < 0.001 | < 0.001 | < 0.001 | < 0.001 | < 0.001 | 0.99998 |  | 0.74544 | 0.87842 |
|  | **Low-NS5** | < 0.001 | < 0.001 | < 0.001 | < 0.001 | < 0.001 | 0.49541 | 0.74544 |  | 0.06818 |
|  | **Low-Con** | < 0.001 | < 0.001 | < 0.001 | < 0.001 | < 0.001 | 0.97895 | 0.87842 | 0.06818 |  |
| **DENV-3** | **P-value** | **CDS** | **Hi-E** | **Hi-NS2A** | **Hi-NS5** | **Hi-Con** | **Low-NS3** | **Low-NS4B** | **Low-NS5** | **Low-Con** |
|  | **CDS** |  | 0.97434 | 0.47723 | 0.99815 | 0.97963 | < 0.001 | < 0.001 | < 0.001 | < 0.001 |
|  | **Hi-E** | 0.97434 |  | 0.98023 | 0.99999 | 1.00000 | < 0.001 | < 0.001 | < 0.001 | < 0.001 |
|  | **Hi-NS2A** | 0.47723 | 0.98023 |  | 0.89595 | 0.97504 | < 0.001 | < 0.001 | < 0.001 | < 0.001 |
|  | **Hi-NS5** | 0.99815 | 0.99999 | 0.89595 |  | 1.00000 | < 0.001 | < 0.001 | < 0.001 | < 0.001 |
|  | **Hi-Con** | 0.97963 | 1.00000 | 0.97504 | 1.00000 |  | < 0.001 | < 0.001 | < 0.001 | < 0.001 |
|  | **Low-NS3** | < 0.001 | < 0.001 | < 0.001 | < 0.001 | < 0.001 |  | 0.16027 | 0.98767 | 0.67580 |
|  | **Low-NS4B** | < 0.001 | < 0.001 | < 0.001 | < 0.001 | < 0.001 | 0.16027 |  | 0.01517 | 0.98928 |
|  | **Low-NS5** | < 0.001 | < 0.001 | < 0.001 | < 0.001 | < 0.001 | 0.98767 | 0.01517 |  | 0.15354 |
|  | **Low-Con** | < 0.001 | < 0.001 | < 0.001 | < 0.001 | < 0.001 | 0.67580 | 0.98928 | 0.15354 |  |
| **DENV-4** | **P-value** | **CDS** | **Hi-E** | **Hi-NS2A** | **Hi-NS5** | **Hi-Con** | **Low-NS3** | **Low-NS4B** | **Low-NS5** | **Low-Con** |
|  | **CDS** |  | 0.98477 | 1.00000 | 1.00000 | 1.00000 | < 0.001 | < 0.001 | < 0.001 | < 0.001 |
|  | **Hi-E** | 0.98477 |  | 0.97933 | 0.98638 | 0.99665 | < 0.001 | < 0.001 | < 0.001 | < 0.001 |
|  | **Hi-NS2A** | 1.00000 | 0.97933 |  | 1.00000 | 1.00000 | < 0.001 | < 0.001 | < 0.001 | < 0.001 |
|  | **Hi-NS5** | 1.00000 | 0.98638 | 1.00000 |  | 1.00000 | < 0.001 | < 0.001 | < 0.001 | < 0.001 |
|  | **Hi-Con** | 1.00000 | 0.99665 | 1.00000 | 1.00000 |  | < 0.001 | < 0.001 | < 0.001 | < 0.001 |
|  | **Low-NS3** | < 0.001 | < 0.001 | < 0.001 | < 0.001 | < 0.001 |  | 0.99044 | 0.99788 | 0.13978 |
|  | **Low-NS4B** | < 0.001 | < 0.001 | < 0.001 | < 0.001 | < 0.001 | 0.99044 |  | 1.00000 | 0.62302 |
|  | **Low-NS5** | < 0.001 | < 0.001 | < 0.001 | < 0.001 | < 0.001 | 0.99788 | 1.00000 |  | 0.50225 |
|  | **Low-Con** | < 0.001 | < 0.001 | < 0.001 | < 0.001 | < 0.001 | 0.13978 | 0.62302 | 0.50225 |  |

**Table S2.** Results of the p-value of the Tukey's one-way ANOVA test on BLD of the trees constructed with sequences of a single genotype. One-way analysis of variance (one-way ANOVA) and the p-value of Tukey's test was observed to verify the existence of statistical differences between the scale factor of the different regions evaluated in recapitulating the CDS phylogeny of sequences of the same genotype. The p-values >0.05 are shaded in gray.

Table S3

| **DENV-1** | **P-value** | **CDS** | **Hi-E** | **Hi-NS2A** | **Hi-NS5** | **Hi-Con** | **Low-NS3** | **Low-NS4B** | **Low-NS5** | **Low-Con** |
| --- | --- | --- | --- | --- | --- | --- | --- | --- | --- | --- |
|  | **CDS** |  | < 0.001 | < 0.001 | < 0.001 | < 0.001 | < 0.001 | < 0.001 | < 0.001 | < 0.001 |
|  | **Hi-E** | < 0.001 |  | 1.00000 | 0.01616 | 0.00152 | 0.00268 | 0.00888 | 0.00152 | 0.55823 |
|  | **Hi-NS2A** | < 0.001 | 1.00000 |  | 0.00913 | 0.00283 | 0.00144 | 0.00492 | < 0.001 | 0.68970 |
|  | **Hi-NS5** | < 0.001 | 0.01616 | 0.00913 |  | < 0.001 | 0.99943 | 1.00000 | 0.99637 | < 0.001 |
|  | **Hi-Con** | < 0.001 | 0.00152 | 0.00283 | < 0.001 |  | < 0.001 | < 0.001 | < 0.001 | 0.26153 |
|  | **Low-NS3** | < 0.001 | 0.00268 | 0.00144 | 0.99943 | < 0.001 |  | 0.99998 | 1.00000 | < 0.001 |
|  | **Low-NS4B** | < 0.001 | 0.00888 | 0.00492 | 1.00000 | < 0.001 | 0.99998 |  | 0.99960 | < 0.001 |
|  | **Low-NS5** | < 0.001 | 0.00152 | < 0.001 | 0.99637 | < 0.001 | 1.00000 | 0.99960 |  | < 0.001 |
|  | **Low-Con** | < 0.001 | 0.55823 | 0.68970 | < 0.001 | 0.26153 | < 0.001 | < 0.001 | < 0.001 |  |
| **DENV-2** | **P-value** | **CDS** | **Hi-E** | **Hi-NS2A** | **Hi-NS5** | **Hi-Con** | **Low-NS3** | **Low-NS4B** | **Low-NS5** | **Low-Con** |
|  | **CDS** |  | < 0.001 | < 0.001 | < 0.001 | < 0.001 | < 0.001 | < 0.001 | < 0.001 | < 0.001 |
|  | **Hi-E** | < 0.001 |  | 0.03992 | 0.99788 | < 0.001 | 0.99940 | 0.51555 | 0.41608 | < 0.001 |
|  | **Hi-NS2A** | < 0.001 | 0.03992 |  | 0.21235 | 0.10973 | 0.00727 | 0.92864 | < 0.001 | 0.03805 |
|  | **Hi-NS5** | < 0.001 | 0.99788 | 0.21235 |  | < 0.001 | 0.90707 | 0.92135 | 0.10362 | < 0.001 |
|  | **Hi-Con** | < 0.001 | < 0.001 | 0.10973 | < 0.001 |  | < 0.001 | 0.00346 | < 0.001 | 0.99995 |
|  | **Low-NS3** | < 0.001 | 0.99940 | 0.00727 | 0.90707 | < 0.001 |  | 0.18627 | 0.80408 | < 0.001 |
|  | **Low-NS4B** | < 0.001 | 0.51555 | 0.92864 | 0.92135 | 0.00346 | 0.18627 |  | 0.00298 | < 0.001 |
|  | **Low-NS5** | < 0.001 | 0.41608 | < 0.001 | 0.10362 | < 0.001 | 0.80408 | 0.00298 |  | < 0.001 |
|  | **Low-Con** | < 0.001 | < 0.001 | 0.03805 | < 0.001 | 0.99995 | < 0.001 | < 0.001 | < 0.001 |  |
| **DENV-3** | **P-value** | **CDS** | **Hi-E** | **Hi-NS2A** | **Hi-NS5** | **Hi-Con** | **Low-NS3** | **Low-NS4B** | **Low-NS5** | **Low-Con** |
|  | **CDS** |  | < 0.001 | < 0.001 | < 0.001 | < 0.001 | < 0.001 | < 0.001 | < 0.001 | < 0.001 |
|  | **Hi-E** | < 0.001 |  | 0.97842 | 0.24246 | < 0.001 | 0.00893 | 0.60333 | < 0.001 | < 0.001 |
|  | **Hi-NS2A** | < 0.001 | 0.97842 |  | 0.85528 | < 0.001 | 0.13046 | 0.99340 | < 0.001 | < 0.001 |
|  | **Hi-NS5** | < 0.001 | 0.24246 | 0.85528 |  | < 0.001 | 0.90722 | 0.99943 | < 0.001 | < 0.001 |
|  | **Hi-Con** | < 0.001 | < 0.001 | < 0.001 | < 0.001 |  | < 0.001 | < 0.001 | < 0.001 | 0.99973 |
|  | **Low-NS3** | < 0.001 | 0.00893 | 0.13046 | 0.90722 | < 0.001 |  | 0.56855 | < 0.001 | < 0.001 |
|  | **Low-NS4B** | < 0.001 | 0.60333 | 0.99340 | 0.99943 | < 0.001 | 0.56855 |  | < 0.001 | < 0.001 |
|  | **Low-NS5** | < 0.001 | < 0.001 | < 0.001 | < 0.001 | < 0.001 | < 0.001 | < 0.001 |  | < 0.001 |
|  | **Low-Con** | < 0.001 | < 0.001 | < 0.001 | < 0.001 | 0.99973 | < 0.001 | < 0.001 | < 0.001 |  |
| **DENV-4** | **P-value** | **CDS** | **Hi-E** | **Hi-NS2A** | **Hi-NS5** | **Hi-Con** | **Low-NS3** | **Low-NS4B** | **Low-NS5** | **Low-Con** |
|  | **CDS** |  | < 0.001 | < 0.001 | < 0.001 | 0.00104 | < 0.001 | < 0.001 | < 0.001 | < 0.001 |
|  | **Hi-E** | < 0.001 |  | < 0.001 | 0.02640 | < 0.001 | 0.25470 | 0.16910 | 0.01945 | < 0.001 |
|  | **Hi-NS2A** | < 0.001 | < 0.001 |  | 0.01542 | 0.29871 | < 0.001 | 0.00152 | 0.02105 | 0.91344 |
|  | **Hi-NS5** | < 0.001 | 0.02640 | 0.01542 |  | < 0.001 | 0.98452 | 0.99684 | 1.00000 | < 0.001 |
|  | **Hi-Con** | 0.00104 | < 0.001 | 0.29871 | < 0.001 |  | < 0.001 | < 0.001 | < 0.001 | 0.97432 |
|  | **Low-NS3** | < 0.001 | 0.25470 | < 0.001 | 0.98452 | < 0.001 |  | 1.00000 | 0.97001 | < 0.001 |
|  | **Low-NS4B** | < 0.001 | 0.16910 | 0.00152 | 0.99684 | < 0.001 | 1.00000 |  | 0.99213 | < 0.001 |
|  | **Low-NS5** | < 0.001 | 0.01945 | 0.02105 | 1.00000 | < 0.001 | 0.97001 | 0.99213 |  | < 0.001 |
|  | **Low-Con** | < 0.001 | < 0.001 | 0.91344 | < 0.001 | 0.97432 | < 0.001 | < 0.001 | < 0.001 |  |

**Table S3.** Results of the p-value of the Tukey's one-way ANOVA test on the topological incongruence of the trees constructed with sequences of different genotypes. One-way analysis of variance (one-way ANOVA) and the p-value of Tukey's test was observed to verify the existence of statistical differences between K-Tree score of the different regions evaluated in recapitulating the CDS phylogeny of sequences of different genotypes. The p-values >0.05 are shaded in gray.

Table S4

| **DENV-1** | **P-value** | **CDS** | **Hi-E** | **Hi-NS2A** | **Hi-NS5** | **Hi-Con** | **Low-NS3** | **Low-NS4B** | **Low-NS5** | **Low-Con** |
| --- | --- | --- | --- | --- | --- | --- | --- | --- | --- | --- |
|  | **CDS** |  | < 0.001 | < 0.001 | < 0.001 | 0.04367 | < 0.001 | < 0.001 | < 0.001 | 0.00580 |
|  | **Hi-E** | < 0.001 |  | 1.00000 | 1.00000 | 0.03746 | 0.82504 | 0.20359 | 0.94557 | 0.23376 |
|  | **Hi-NS2A** | < 0.001 | 1.00000 |  | 1.00000 | 0.04732 | 0.77621 | 0.16957 | 0.91972 | 0.27621 |
|  | **Hi-NS5** | < 0.001 | 1.00000 | 1.00000 |  | 0.04911 | 0.76783 | 0.16451 | 0.91491 | 0.28350 |
|  | **Hi-Con** | 0.04367 | 0.03746 | 0.04732 | 0.04911 |  | 0.00038 | < 0.001 | 0.00106 | 0.99584 |
|  | **Low-NS3** | < 0.001 | 0.82504 | 0.77621 | 0.76783 | 0.00038 |  | 0.97455 | 1.00000 | 0.00479 |
|  | **Low-NS4B** | < 0.001 | 0.20359 | 0.16957 | 0.16451 | < 0.001 | 0.97455 |  | 0.89190 | < 0.001 |
|  | **Low-NS5** | < 0.001 | 0.94557 | 0.91972 | 0.91491 | 0.00106 | 1.00000 | 0.89190 |  | 0.01221 |
|  | **Low-Con** | 0.00580 | 0.23376 | 0.27621 | 0.28350 | 0.99584 | 0.00479 | < 0.001 | 0.01221 |  |
| **DENV-2** | **P-value** | **CDS** | **Hi-E** | **Hi-NS2A** | **Hi-NS5** | **Hi-Con** | **Low-NS3** | **Low-NS4B** | **Low-NS5** | **Low-Con** |
|  | **CDS** |  | < 0.001 | < 0.001 | < 0.001 | 0.04135 | < 0.001 | < 0.001 | < 0.001 | 0.00122 |
|  | **Hi-E** | < 0.001 |  | 1.00000 | 1.00000 | 0.02567 | 0.99974 | 0.94989 | 0.65068 | 0.36748 |
|  | **Hi-NS2A** | < 0.001 | 1.00000 |  | 1.00000 | 0.03267 | 0.99921 | 0.92550 | 0.59096 | 0.42180 |
|  | **Hi-NS5** | < 0.001 | 1.00000 | 1.00000 |  | 0.03660 | 0.99873 | 0.91145 | 0.56208 | 0.44900 |
|  | **Hi-Con** | 0.04135 | 0.02567 | 0.03267 | 0.03660 |  | 0.00540 | < 0.001 | < 0.001 | 0.94734 |
|  | **Low-NS3** | < 0.001 | 0.99974 | 0.99921 | 0.99873 | 0.00540 |  | 0.99906 | 0.92717 | 0.13060 |
|  | **Low-NS4B** | < 0.001 | 0.94989 | 0.92550 | 0.91145 | < 0.001 | 0.99906 |  | 0.99926 | 0.02642 |
|  | **Low-NS5** | < 0.001 | 0.65068 | 0.59096 | 0.56208 | < 0.001 | 0.92717 | 0.99926 |  | 0.00432 |
|  | **Low-Con** | 0.00122 | 0.36748 | 0.42180 | 0.44900 | 0.94734 | 0.13060 | 0.02642 | 0.00432 |  |
| **DENV-3** | **P-value** | **CDS** | **Hi-E** | **Hi-NS2A** | **Hi-NS5** | **Hi-Con** | **Low-NS3** | **Low-NS4B** | **Low-NS5** | **Low-Con** |
|  | **CDS** |  | < 0.001 | < 0.001 | < 0.001 | 0.00262 | < 0.001 | < 0.001 | < 0.001 | < 0.001 |
|  | **Hi-E** | < 0.001 |  | 0.99818 | 0.63386 | 0.04581 | 0.99999 | 1.00000 | 0.97305 | 0.56063 |
|  | **Hi-NS2A** | < 0.001 | 0.99818 |  | 0.96239 | 0.22842 | 0.97093 | 0.97803 | 0.67461 | 0.93680 |
|  | **Hi-NS5** | < 0.001 | 0.63386 | 0.96239 |  | 0.88516 | 0.39075 | 0.42082 | 0.10077 | 1.00000 |
|  | **Hi-Con** | 0.00262 | 0.04581 | 0.22842 | 0.88516 |  | 0.01681 | 0.01927 | 0.00210 | 0.92432 |
|  | **Low-NS3** | < 0.001 | 0.99999 | 0.97093 | 0.39075 | 0.01681 |  | 1.00000 | 0.99841 | 0.32752 |
|  | **Low-NS4B** | < 0.001 | 1.00000 | 0.97803 | 0.42082 | 0.01927 | 1.00000 |  | 0.99744 | 0.35511 |
|  | **Low-NS5** | < 0.001 | 0.97305 | 0.67461 | 0.10077 | 0.00210 | 0.99841 | 0.99744 |  | 0.07793 |
|  | **Low-Con** | < 0.001 | 0.56063 | 0.93680 | 1.00000 | 0.92432 | 0.32752 | 0.35511 | 0.07793 |  |
| **DENV-4** | **P-value** | **CDS** | **Hi-E** | **Hi-NS2A** | **Hi-NS5** | **Hi-Con** | **Low-NS3** | **Low-NS4B** | **Low-NS5** | **Low-Con** |
|  | **CDS** |  | < 0.001 | < 0.001 | < 0.001 | < 0.001 | < 0.001 | < 0.001 | < 0.001 | < 0.001 |
|  | **Hi-E** | < 0.001 |  | 0.98996 | 0.42949 | 0.82936 | 0.81627 | 0.80418 | 0.99930 | 0.00424 |
|  | **Hi-NS2A** | < 0.001 | 0.98996 |  | 0.07005 | 0.27269 | 0.26030 | 0.24955 | 0.81959 | < 0.001 |
|  | **Hi-NS5** | < 0.001 | 0.42949 | 0.07005 |  | 0.99917 | 0.99939 | 0.99954 | 0.82194 | 0.57818 |
|  | **Hi-Con** | < 0.001 | 0.82936 | 0.27269 | 0.99917 |  | 1.00000 | 1.00000 | 0.99126 | 0.21263 |
|  | **Low-NS3** | < 0.001 | 0.81627 | 0.26030 | 0.99939 | 1.00000 |  | 1.00000 | 0.98949 | 0.22343 |
|  | **Low-NS4B** | < 0.001 | 0.80418 | 0.24955 | 0.99954 | 1.00000 | 1.00000 |  | 0.98770 | 0.23340 |
|  | **Low-NS5** | < 0.001 | 0.99930 | 0.81959 | 0.82194 | 0.99126 | 0.98949 | 0.98770 |  | 0.02557 |
|  | **Low-Con** | < 0.001 | 0.00424 | < 0.001 | 0.57818 | 0.21263 | 0.22343 | 0.23340 | 0.02557 |  |

**Table S4.** Results of the p-value of the Tukey's one-way ANOVA test on the PP mean of the trees constructed with sequences of a single genotype. One-way analysis of variance (one-way ANOVA) and the p-value of Tukey's test was observed to verify the existence of statistical differences between K-Tree score of the different regions evaluated in recapitulating the CDS phylogeny of sequences of the same genotype. The p-values >0.05 are shaded in gray.

Table S5

| **DENV-1** | **P-value** | **CDS** | **Hi-E** | **Hi-NS2A** | **Hi-NS5** | **Hi-Con** | **Low-NS3** | **Low-NS4B** | **Low-NS5** | **Low-Con** |
| --- | --- | --- | --- | --- | --- | --- | --- | --- | --- | --- |
|  | **CDS** |  | < 0.001 | < 0.001 | < 0.001 | 0.41 | < 0.001 | < 0.001 | 0.01 | 0.33 |
|  | **Hi-E** | < 0.001 |  | 1.00 | 1.00 | 0.09 | 0.89 | 0.05 | 0.89 | 0.12 |
|  | **Hi-NS2A** | < 0.001 | 1.00 |  | 0.97 | 0.27 | 0.60 | 0.01 | 0.99 | 0.33 |
|  | **Hi-NS5** | < 0.001 | 1.00 | 0.97 |  | 0.02 | 1.00 | 0.19 | 0.55 | 0.03 |
|  | **Hi-Con** | 0.41 | 0.09 | 0.27 | 0.02 |  | < 0.001 | < 0.001 | 0.80 | 1.00 |
|  | **Low-NS3** | < 0.001 | 0.89 | 0.60 | 1.00 | < 0.001 |  | 0.65 | 0.14 | < 0.001 |
|  | **Low-NS4B** | < 0.001 | 0.05 | 0.01 | 0.19 | < 0.001 | 0.65 |  | < 0.001 | < 0.001 |
|  | **Low-NS5** | 0.01 | 0.89 | 0.99 | 0.55 | 0.80 | 0.14 | < 0.001 |  | 0.86 |
|  | **Low-Con** | 0.33 | 0.12 | 0.33 | 0.03 | 1.00 | < 0.001 | < 0.001 | 0.86 |  |
| **DENV-2** | **P-value** | **CDS** | **Hi-E** | **Hi-NS2A** | **Hi-NS5** | **Hi-Con** | **Low-NS3** | **Low-NS4B** | **Low-NS5** | **Low-Con** |
|  | **CDS** |  | < 0.001 | < 0.001 | < 0.001 | 0.03 | 0.00 | < 0.001 | < 0.001 | 0.01 |
|  | **Hi-E** | < 0.001 |  | 1.00 | 0.99 | < 0.001 | 0.99 | 0.04 | 0.99 | 0.02 |
|  | **Hi-NS2A** | < 0.001 | 1.00 |  | 1.00 | 0.01 | 0.96 | 0.02 | 0.97 | 0.04 |
|  | **Hi-NS5** | < 0.001 | 0.99 | 1.00 |  | 0.04 | 0.70 | < 0.001 | 0.74 | 0.17 |
|  | **Hi-Con** | 0.03 | < 0.001 | 0.01 | 0.04 |  | < 0.001 | < 0.001 | < 0.001 | 1.00 |
|  | **Low-NS3** | 0.00 | 0.99 | 0.96 | 0.70 | < 0.001 |  | 0.29 | 1.00 | < 0.001 |
|  | **Low-NS4B** | < 0.001 | 0.04 | 0.02 | < 0.001 | < 0.001 | 0.29 |  | 0.26 | < 0.001 |
|  | **Low-NS5** | < 0.001 | 0.99 | 0.97 | 0.74 | < 0.001 | 1.00 | 0.26 |  | < 0.001 |
|  | **Low-Con** | 0.01 | 0.02 | 0.04 | 0.17 | 1.00 | < 0.001 | < 0.001 | < 0.001 |  |
| **DENV-3** | **P-value** | **CDS** | **Hi-E** | **Hi-NS2A** | **Hi-NS5** | **Hi-Con** | **Low-NS3** | **Low-NS4B** | **Low-NS5** | **Low-Con** |
|  | **CDS** |  | < 0.001 | < 0.001 | < 0.001 | < 0.001 | < 0.001 | < 0.001 | < 0.001 | < 0.001 |
|  | **Hi-E** | < 0.001 |  | < 0.001 | < 0.001 | < 0.001 | 1.00 | < 0.001 | < 0.001 | < 0.001 |
|  | **Hi-NS2A** | < 0.001 | < 0.001 |  | < 0.001 | < 0.001 | < 0.001 | 0.98 | 0.96 | < 0.001 |
|  | **Hi-NS5** | < 0.001 | < 0.001 | < 0.001 |  | 0.93 | < 0.001 | 0.01 | 0.02 | 1.00 |
|  | **Hi-Con** | < 0.001 | < 0.001 | < 0.001 | 0.93 |  | < 0.001 | < 0.001 | < 0.001 | 0.79 |
|  | **Low-NS3** | < 0.001 | 1.00 | < 0.001 | < 0.001 | < 0.001 |  | < 0.001 | < 0.001 | < 0.001 |
|  | **Low-NS4B** | < 0.001 | < 0.001 | 0.98 | 0.01 | < 0.001 | < 0.001 |  | 1.00 | 0.03 |
|  | **Low-NS5** | < 0.001 | < 0.001 | 0.96 | 0.02 | < 0.001 | < 0.001 | 1.00 |  | 0.04 |
|  | **Low-Con** | < 0.001 | < 0.001 | < 0.001 | 1.00 | 0.79 | < 0.001 | 0.03 | 0.04 |  |
| **DENV-4** | **P-value** | **CDS** | **Hi-E** | **Hi-NS2A** | **Hi-NS5** | **Hi-Con** | **Low-NS3** | **Low-NS4B** | **Low-NS5** | **Low-Con** |
|  | **CDS** |  | < 0.001 | < 0.001 | < 0.001 | < 0.001 | < 0.001 | < 0.001 | < 0.001 | < 0.001 |
|  | **Hi-E** | < 0.001 |  | 0.08 | 0.02 | 0.08 | 0.11 | 0.58 | 0.95 | < 0.001 |
|  | **Hi-NS2A** | < 0.001 | 0.08 |  | 1.00 | < 0.001 | 1.00 | 0.97 | 0.63 | < 0.001 |
|  | **Hi-NS5** | < 0.001 | 0.02 | 1.00 |  | < 0.001 | < 0.001 | < 0.001 | 0.95 |  |
|  | **Hi-Con** | < 0.001 | 0.08 | < 0.001 | < 0.001 |  | 0.99 | 0.73 | < 0.001 |  |
|  | **Low-NS3** | < 0.001 | 0.11 | 1.00 | 1.00 | < 0.001 |  | 1.00 | < 0.001 |  |
|  | **Low-NS4B** | < 0.001 | 0.58 | 0.97 | 0.73 | < 0.001 | 0.99 |  | 1.00 | < 0.001 |
|  | **Low-NS5** | < 0.001 | 0.95 | 0.63 | 0.27 | < 0.001 | 0.73 | 1.00 |  | < 0.001 |
|  | **Low-Con** | < 0.001 | < 0.001 | < 0.001 | < 0.001 | 0.95 | < 0.001 | < 0.001 | < 0.001 |  |

**Table S5.** Results of the p-value of the Tukey's one-way ANOVA test on tree confidence of the trees constructed with sequences of different genotypes. One-way analysis of variance (one-way ANOVA) and the p-value of Tukey's test was observed to verify the existence of statistical differences between the support values of the different regions evaluated in recapitulating the CDS phylogeny of sequences of different genotypes. The p-values >0.05 are shaded in gray.

Table S6

| **DENV-1** | **P-value** | **CDS** | **Hi-E** | **Hi-NS2A** | **Hi-NS5** | **Hi-Con** | **Low-NS3** | **Low-NS4B** | **Low-NS5** | **Low-Con** |
| --- | --- | --- | --- | --- | --- | --- | --- | --- | --- | --- |
|  | **CDS** |  | < 0.001 | < 0.001 | < 0.001 | 0.22852 | < 0.001 | < 0.001 | < 0.001 | < 0.001 |
|  | **Hi-E** | < 0.001 |  | 0.47212 | 0.99719 | 0.00934 | 0.10312 | < 0.001 | < 0.001 | 0.97409 |
|  | **Hi-NS2A** | < 0.001 | 0.47212 |  | 0.90920 | 0.70840 | < 0.001 | < 0.001 | < 0.001 | 0.97947 |
|  | **Hi-NS5** | < 0.001 | 0.99719 | 0.90920 |  | 0.00716 | < 0.001 | < 0.001 | < 0.001 | 1.00000 |
|  | **Hi-Con** | 0.22852 | 0.00934 | 0.70840 | 0.07157 |  | < 0.001 | < 0.001 | < 0.001 | 0.14445 |
|  | **Low-NS3** | < 0.001 | 0.10312 | < 0.001 | < 0.001 | < 0.001 |  | 0.09995 | 0.93336 | 0.00596 |
|  | **Low-NS4B** | < 0.001 | < 0.001 | < 0.001 | < 0.001 | < 0.001 | 0.09995 |  | 0.74847 | < 0.001 |
|  | **Low-NS5** | < 0.001 | < 0.001 | < 0.001 | < 0.001 | < 0.001 | 0.93336 | 0.74847 |  | < 0.001 |
|  | **Low-Con** | < 0.001 | 0.97409 | 0.97947 | 1.00000 | 0.01445 | 0.00596 | < 0.001 | < 0.001 |  |
| **DENV-2** | **P-value** | **CDS** | **Hi-E** | **Hi-NS2A** | **Hi-NS5** | **Hi-Con** | **Low-NS3** | **Low-NS4B** | **Low-NS5** | **Low-Con** |
|  | **CDS** |  | < 0.001 | < 0.001 | < 0.001 | 0.25755 | < 0.001 | < 0.001 | < 0.001 | < 0.001 |
|  | **Hi-E** | < 0.001 |  | 0.99995 | 0.31725 | 0.23933 | < 0.001 | < 0.001 | < 0.001 | 0.35583 |
|  | **Hi-NS2A** | < 0.001 | 0.99995 |  | 0.13484 | 0.48779 | < 0.001 | < 0.001 | < 0.001 | 0.15639 |
|  | **Hi-NS5** | < 0.001 | 0.31725 | 0.13484 |  | < 0.001 | < 0.001 | < 0.001 | 0.11772 | 1.00000 |
|  | **Hi-Con** | 0.25755 | 0.23933 | 0.48779 | < 0.001 |  | < 0.001 | < 0.001 | < 0.001 | < 0.001 |
|  | **Low-NS3** | < 0.001 | < 0.001 | < 0.001 | < 0.001 | < 0.001 |  | 0.98990 | < 0.001 | < 0.001 |
|  | **Low-NS4B** | < 0.001 | < 0.001 | < 0.001 | < 0.001 | < 0.001 | 0.98990 |  | 0.00525 | < 0.001 |
|  | **Low-NS5** | < 0.001 | < 0.001 | < 0.001 | 0.11772 | < 0.001 | < 0.001 | 0.00525 |  | 0.10063 |
|  | **Low-Con** | < 0.001 | 0.35583 | 0.15639 | 1.00000 | < 0.001 | < 0.001 | < 0.001 | 0.10063 |  |
| **DENV-3** | **P-value** | **CDS** | **Hi-E** | **Hi-NS2A** | **Hi-NS5** | **Hi-Con** | **Low-NS3** | **Low-NS4B** | **Low-NS5** | **Low-Con** |
|  | **CDS** |  | < 0.001 | 0.01 | < 0.001 | 0.14 | < 0.001 | < 0.001 | < 0.001 | < 0.001 |
|  | **Hi-E** | < 0.001 |  | 0.91 | 0.83 | 0.24 | < 0.001 | < 0.001 | < 0.001 | 0.87 |
|  | **Hi-NS2A** | 0.01 | 0.91 |  | 0.12 | 0.95 | < 0.001 | < 0.001 | < 0.001 | 0.15 |
|  | **Hi-NS5** | < 0.001 | 0.83 | 0.12 |  | 0.01 | < 0.001 | < 0.001 | < 0.001 | 1.00 |
|  | **Hi-Con** | 0.14 | 0.24 | 0.95 | 0.01 |  | < 0.001 | < 0.001 | < 0.001 | 0.01 |
|  | **Low-NS3** | < 0.001 | < 0.001 | < 0.001 | < 0.001 | < 0.001 |  | 1.00 | 0.15 | < 0.001 |
|  | **Low-NS4B** | < 0.001 | < 0.001 | < 0.001 | < 0.001 | < 0.001 | 1.00 |  | 0.15 | < 0.001 |
|  | **Low-NS5** | < 0.001 | < 0.001 | < 0.001 | < 0.001 | < 0.001 | 0.15 | 0.15 |  | < 0.001 |
|  | **Low-Con** | < 0.001 | 0.87 | 0.15 | 1.00 | 0.01 | < 0.001 | < 0.001 | < 0.001 |  |
| **DENV-4** | **P-value** | **CDS** | **Hi-E** | **Hi-NS2A** | **Hi-NS5** | **Hi-Con** | **Low-NS3** | **Low-NS4B** | **Low-NS5** | **Low-Con** |
|  | **CDS** |  | < 0.001 | 0.01 | < 0.001 | 0.11 | < 0.001 | < 0.001 | < 0.001 | < 0.001 |
|  | **Hi-E** | < 0.001 |  | 0.97 | 0.90 | 0.43 | < 0.001 | < 0.001 | < 0.001 | 0.79 |
|  | **Hi-NS2A** | 0.01 | 0.97 |  | 0.26 | 0.97 | < 0.001 | < 0.001 | < 0.001 | 0.17 |
|  | **Hi-NS5** | < 0.001 | 0.90 | 0.26 |  | 0.02 | < 0.001 | < 0.001 | 0.01 | 1.00 |
|  | **Hi-Con** | 0.11 | 0.43 | 0.97 | 0.02 |  | < 0.001 | < 0.001 | < 0.001 | 0.01 |
|  | **Low-NS3** | < 0.001 | < 0.001 | < 0.001 | < 0.001 | < 0.001 |  | 1.00 | 0.14 | < 0.001 |
|  | **Low-NS4B** | < 0.001 | < 0.001 | < 0.001 | < 0.001 | < 0.001 | 1.00 |  | 0.36 | < 0.001 |
|  | **Low-NS5** | < 0.001 | < 0.001 | < 0.001 | 0.01 | < 0.001 | 0.14 | 0.36 |  | 0.01 |
|  | **Low-Con** | < 0.001 | 0.79 | 0.17 | 1.00 | 0.01 | < 0.001 | < 0.001 | 0.01 |  |

**Table S6.** Results of the p-value of the Tukey's one-way ANOVA test on tree confidence of the trees constructed with sequences of a single genotype. One-way analysis of variance (one-way ANOVA) and the p-value of Tukey's test was observed to verify the existence of statistical differences between the support values of the different regions evaluated in recapitulating the CDS phylogeny of sequences of the same genotype. The p-values >0.05 are shaded in gray.

Table S7

a)

b)

| **Different Genotype** | **DENV-1** | | | **DENV-2** | | | **DENV-3** | | | **DENV-4** | | |
| --- | --- | --- | --- | --- | --- | --- | --- | --- | --- | --- | --- | --- |
|  | **Scale Factor (SD)** | **K-score (SD)** | **Mean PP (SD)** | **Scale Factor (SD)** | **K-score (SD)** | **Mean PP (SD)** | **Scale Factor (SD)** | **K-score (SD)** | **Mean PP (SD)** | **Scale Factor (SD)** | **K-score (SD)** | **Mean PP (SD)** |
| **CDS** | 1 (0) | 0 (0) | 0.99 (0.01) | 1 (0) | 0 (0) | 0.99 (0.01) | 1 (0) | 0 (0) | 0.98 (0.01) | 1 (0) | 0 (0) | 0.98 (0.01) |
| **Hi-E** | 0.92 (0.05) | 0.028 (0.004) | 0.89 (0.05) | 1.41 (0.12) | 0.091 (0.018) | 0.86 (0.04) | 0.92 (0.05) | 0.025 (0.001) | 0.76 (0.03) | 1.04 (0.14) | 0.459 (0.086) | 0.83 (0.01) |
| **Hi-NS2A** | 0.79 (0.05) | 0.027 (0.011) | 0.9 (0.03) | 1.04 (0.06) | 0.067 (0.017) | 0.87 (0.03) | 0.79 (0.04) | 0.027 (0.003) | 0.83 (0.02) | 0.86 (0.13) | 0.226 (0.05) | 0.79 (0.03) |
| **Hi-NS5** | 0.88 (0.06) | 0.037 (0.004) | 0.88 (0.02) | 1.25 (0.03) | 0.086 (0.009) | 0.88 (0.05) | 0.88 (0.05) | 0.029 (0.001) | 0.89 (0.02) | 1.1 (0.2) | 0.346 (0.52) | 0.78 (0.04) |
| **Hi-Con** | 0.80 (0.04) | 0.016 (0.001) | 0.95 (0.01) | 0.98 (0.03) | 0.046 (0.01) | 0.93 (0.02) | 0.79 (0.03) | 0.018 (0.001) | 0.91 (0.03) | 0.96 (0.07) | 0.148 (0.045) | 0.87 (0.04) |
| **Low-NS3** | 1.1 (0.06) | 0.039 (0.004) | 0.87 (0.05) | 1.57 (0.04) | 0.096 (0.01) | 0.85 (0.03) | 1.12 (0.04) | 0.031 (0.004) | 0.76 (0.02) | 2.06 (0.29) | 0.378 (0.074) | 0.79 (0.02) |
| **Low-NS4B** | 1.06 (0.08) | 0.038 (0.003) | 0.83 (0.03) | 1.43 (0.13) | 0.076 (0.011) | 0.81 (0.01) | 1.06 (0.8) | 0.028(0.003) | 0.84 (0.05) | 1.48 (0.18) | 0.371 (0.066) | 0.8 (0.02) |
| **Low-NS5** | 1.2 (0.07 | 0.039 (0.002) | 0.92 (0.04) | 1.8 (0.21) | 0.107 (0.019) | 0.85 (0.03) | 1.01 (0.1) | 0.038 (0.003) | 0.84 (0.02) | 1.66 (0.28) | 0.342 (0.047) | 0.81 (0.02) |
| **Low-Con** | 1.12 (0.06) | 0.023 (0.002) | 0.95 (0.03) | 1.3 (0.03) | 0.042 (0.005) | 0.92 (0.02) | 1.05 (0.05) | 0.018 (0.001) | 0.89 (0.01) | 1.1 (0.13) | 0.183 (0.037) | 0.89 (0.02) |
|  |  |  |  |  |  |  |  |  |  |  |  |  |
|  |  |  |  |  |  |  |  |  |  |  |  |  |
|  |  |  |  |  |  |  |  |  |  |  |  |  |
| **Same Genotype** | **DENV-1** | | | **DENV-2** | | | **DENV-3** | | | **DENV-4** | | |
|  | **Scale Factor (SD)** | **K-score (SD)** | **Mean PP (SD)** | **Scale Factor (SD)** | **K-score (SD)** | **Mean PP (SD)** | **Scale Factor (SD)** | **K-score (SD)** | **Mean PP (SD)** | **Scale Factor (SD)** | **K-score (SD)** | **Mean PP (SD)** |
| **CDS** | 1 (0) | 0 (0) | 0.99 (0.01) | 1 (0) | 0 (0) | 0.98 (0.01) | 1 (0) | 0 (0) | 0.97 (0.01) | 1 (0) | 0 (0) | 0.98 (0.01) |
| **Hi-E** | 0.16 (0.1) | 0.021 (0.003) | 0.83 (0.05) | 1.08 (0.14) | 0.018 (0.004) | 0.88 (0.03) | 0.8 (0.13) | 0.023 (0.005) | 0.86 (0.04) | 0.73 (0.12) | 0.017 (0.004) | 0.86 (0.05) |
| **Hi-NS2A** | 0.89 (0.1) | 0.02 (0.004) | 0.88 (0.04) | 0.71 (0.08) | 0.018 (0.004) | 0.89 (0.05) | 0.6 (0.17) | 0.021 (0.005) | 0.89 (0.03) | 1.01 (0.16) | 0.019 (0.003) | 0.89 (0.05) |
| **Hi-NS5** | 0.98 (0.07) | 0.02 (0.004) | 0.85 (0.03) | 0.87 (0.09) | 0.018 (0.005) | 0.83 (0.03) | 0.86 (0.15) | 0.017 (0.003) | 0.83 (0.03) | 1 (0.16) | 0.013 (0.003) | 0.83 (0.02) |
| **Hi-Con** | 1.12 (0.07) | 0.01 (0.002) | 0.92 (0.04) | 0.99 (0.11) | 0.009 (0.002) | 0.93 (0.04) | 0.81 (0.1) | 0.0131 (0.004) | 0.91 (0.04) | 0.94 (0.11) | 0.015 (0.005) | 0.91 (0.05) |
| **Low-NS3** | 2.2 (0.43) | 0.025 (0.01) | 0.75 (0.08) | 2.32 (0.38) | 0.02 (0.005) | 0.67 (0.04) | 2.55 (0.41) | 0.024 (0.007) | 0.68 (0.03) | 3.78 (0.59) | 0.015 (0.002) | 0.68 (0.04) |
| **Low-NS4B** | 2.31 (0.36) | 0.029 (0.008) | 0.68 (0.04) | 2.38 (0.22) | 0.022 (0.008) | 0.68 (0.04) | 2.04 (0.6) | 0.024 (0.006) | 0.68 (0.04) | 3.53 (1) | 0.015 (0.002) | 0.69 (0.03) |
| **Low-NS5** | 2.05 (0.13) | 0.024 (0.006) | 0.72 (0.03) | 2.66 (0.62) | 0.023 (0.006) | 0.77 (0.04) | 2.73 (0.52) | 0.026 (0.008) | 0.73 (0.05) | 3.58 (0.68) | 0.016 (0.003) | 0.74 (0.04) |
| **Low-Con** | 1.78 (0.15) | 0.013 (0.002) | 0.85 (0.04) | 2.14 (0.35) | 0.012 (0.003) | 0.83 (0.03) | 2.21 (0.27) | 0.017 (0.005) | 0.83 (0.03) | 3.01 (0.31) | 0.01 (0.002) | 0.83 (0.03) |

**Table S7.** Mean and standard deviation (SD) of the values obtained for Scale factor, K-score and mean PP of the trees constructed with each of the regions evaluated for **a)** sequences of different genotypes and **b)** sequences of the same genotype.
